# Laser-Patterned Alumina Mask and Mask-Less Dry Etch of Si for Light Trapping with Photonic Crystal Structures

**DOI:** 10.3390/mi14030550

**Published:** 2023-02-26

**Authors:** Jovan Maksimovic, Haoran Mu, Daniel Smith, Tomas Katkus, Mantas Vaičiulis, Ramūnas Aleksiejūnas, Gediminas Seniutinas, Soon Hock Ng, Saulius Juodkazis

**Affiliations:** 1 Optical Sciences Centre and Australian Research Council (ARC) Industrial Transformation Training Centre in Surface Engineering for Advanced Materials (SEAM), Swinburne University of Technology, Hawthorn, VIC 3122, Australia; 2 Institute of Photonics and Nanotechnology, Vilnius University, Saulėtekio Ave. 3, 10257 Vilnius, Lithuania; 3 Optical Sciences Centre, School of Science, Swinburne University of Technology, Hawthorn, VIC 3122, Australia; 4 Melbourne Centre for Nanofabrication, 151 Wellington Road, Clayton, VIC 3168, Australia; 5 WRH Program International Research Frontiers Initiative (IRFI), Tokyo Institute of Technology, Nagatsuta-cho, Midori-ku, Yokohama 226-8503, Kanagawa, Japan

**Keywords:** light trapping, Si solar cells, ablation, dry etch, Lambertian limit

## Abstract

Ultra-short 230 fs laser pulses of a 515 nm wavelength were tightly focused onto 700 nm focal spots and utilised in opening ∼0.4–1 μm holes in alumina Al_2_O_3_ etch masks with a 20–50 nm thickness. Such dielectric masks simplify the fabrication of photonic crystal (PhC) light-trapping patterns for the above-Lambertian performance of high-efficiency solar cells. The conditions of the laser ablation of transparent etch masks and the effects sub-surface Si modifications were revealed by plasma etching, numerical modelling, and minority carrier lifetime measurements. Mask-less patterning of Si is proposed using fs laser direct writing for dry plasma etching of Si.

## 1. Introduction

Large-area solar cells have become increasingly important in today’s strive towards a cleaner, greener, renewable energy source. Solar cells are the ultimate large-area application where even the smallest improvement to the solar-to-electrical efficiency can bring significant increases in sustainability and energy efficiency targets [1,2,3,4,5]. Cell efficiency is considered the critical parameter driving commercial development for industrial applications and, consequently, is the focus of most research in this area. Most research on solar cells is targeted towards approaching the theoretical Shockley–Queisser (SQ) light-to-electrical conversion efficiency limit of 33.5% for Si [6]. Life-Cycle Assessment (LCA) assesses the environmental impact of energy generation solutions based on greenhouse gas emissions created over the life-cycle of the energy source. Monocrystalline-Si-based solar cells are made from a highly abundant material, but require significant energy for purification and crystallisation; as such, they have one of the highest environmental impacts of all solar technologies, 29.0–671.0 g CO_2_eq/kWh [7]. This, however, is still much lower than coal (750–1050 g CO_2_eq/kWh), and with Si being the most-mature solar technology, any increase in efficiency contributes to reducing the environmental impact. This is particularly important for the current state of climate change, which was defined as “code red for humanity” in the last Intergovernmental Panel on Climate Change (IPCC) Working Group 1 report on the physical science basis (August 2021). The need to reduce emissions and wean humanity off fossil fuels requires investments into every facet of renewable energy generation.

Before new solar cell designs become ready for industrial upscaling, improvements of the technology have to be independently tested on the centimetre scale at certified labs. Hence, a fs laser direct write of an etch mask in a Cr film of tens of nanometres in thickness was demonstrated for large-area, 2×2 cm^2^, Si solar cell surface texturing for photonic crystal (PhC) patterns [8]. Dry or wet etching of inverse pyramidal (or tee-pee) patterns were carried out through sub-1 μm openings in the mask. It was theoretically demonstrated [9,10,11,12,13,14,15] and experimentally validated [16] that a PhC light trapping is required to break Lambertian (ray optics) light trapping for solar-to-electrical power conversion efficiencies above current record efficiencies around 27% and approaching the single junction limit for Si at ∼32% (one Sun) [17]. The world record equalling an efficiency of ∼25% was demonstrated (Panasonic R&D) using a simple low-temperature passivation via hydrogenation of amorphous Si (a-Si:H) in the heterojunction. In September 2016, Kaneka Ltd., (Osaka, Japan). reported a new record of 26.33% [18], with an interdigitated back contact (IBC) design, delivering record high performance with a surface of random-pyramids for light trapping using 300 μm-thick Si solar cells.

**Figure 1:**
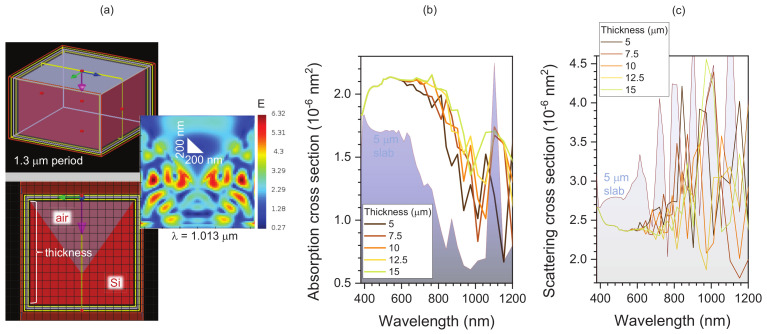
Numerical modelling of light trapping by photonic crystal (PhC) on Si. (**a**) Numerical model and a central cross-section of light enhancement in 1.3 μm-thick Si; period of PhC Λ = 1.3 μm. Calculated by finite difference time domain (FDTD) (Lumerical, Ansys). (**b**) Absorption cross-section $\sigma_{ab}$ of Si of different thicknesses for PhC Λ = 1.3 μm. The background spectral profile is for a flat slab of Si of 5 μm. (**c**) Corresponding scattering cross-sections $\sigma_{sc}$. The indirect Si bandgap at 300 K is 1.12 eV or a 1.107 μm wavelength.

Light-trapping PhC patterns convert incoming sunlight, which is normal to the surface, into in-plane modes and promote absorption even in micro-thin Si (or any other light-absorbing material/composite). Figure 1a illustrates light field enhancement inside a ∼1.3 μm-thick Si solar cell with a light-trapping inverse-pyramidal pattern with period of Λ = 1.3 μm at a wavelength of ∼1 μm close to the absorption edge. The wave nature of light via interference of the slab modes is harnessed for increased absorption. Absorption and scattering cross-sections $\sigma_{ab.sc}$ are shown in Figure 1b,c, respectively. The difference in the absorption of a 5 μm-thick unpatterned slab of Si and textured by PhC is highlighted in (b) and can reach more than 50%. Over the range of a 5 to 15 μm thickness, there is distinct and constant increase of absorption with Si thickness at the longer wavelengths close to the bandgap edge of ∼1 μm. This increase in absorption is aligned with reduced scattering (reflection is accounted for in $\sigma_{sc}$). This qualitative illustration of PhC light trapping in Si has been demonstrated experimentally in PhC surface patterns fabricated using electron-beam-lithography (EBL)-defined etch masks with rectangular openings in Si_3_N_4_ mask [16]. Utilising a Si-on-insulator (SOI) with ∼10 μm-thick Si device layer samples, the Lambertian limit was surpassed at the longer wavelength range where the improvement of light trapping can bring the greatest increase in solar cell efficiency; it was demonstrated over 1×1 cm^2^ area patterns. This demonstration of superior optical light absorption invites the next challenge to apply it to real solar Si cells and fabrication over larger areas, since robust Si_3_N_4_ masks that can withstand etching require high-temperature growth above 250 °C using chemical vapour deposition (CVD).

One of the most-promising geometries of high-efficiency Si solar cells is IBC cells, where surface patterning by PhC is the most easy to incorporate into the fabrication sequence [19,20]. After the steps of doping and the integration of contacts, sealing of the back-side with cross-linked SU8 resist, which can withstand a sequence of wet and plasma etch steps, including piranha and HF solution treatments if required, has to be developed next. EBL or direct laser writing of etch masks can be used where the latter is more practical, since it does not require a vacuum and can be applied over large surface areas. Plasma etching using laser-ablated circular micro-holes is faster than KOH wet etching by an order of magnitude. Plasma etching is another necessary development for the thinning of Si IBC cells to the thickness range where light trapping by PhC patterns is the most promising, i.e., 10–30 μm from typical 190–290 μm Si cells currently developed. At the achievable plasma etching rate of Si at ∼(1–2) μm/min, a practically achievable 1–2 h process time will be required. Most importantly, the surface height distribution over the etched areas is of paramount importance. In this respect, direct laser writing of the etch mask is less sensitive to the micrometre-scale unevenness of the surface height as compared to the more demanding EBL approach. Investigations were carried out in this study to address some of the next challenges in laser mask writing over large areas and developing masks that are not based on metals (e.g., Cr).

Here, different conditions of the laser mask definition by ablation were tested for different thicknesses of alumina (Al_2_O_3_) masks and plasma etching conditions over areas with centimetres in the cross-section. Dielectric etch masks using alumina films compatible with the antireflection coatings of IBC cells, as well as mask-less etching of Si were tested experimentally and by numerical modelling. Changes of the minority carrier lifetime were monitored during the processing steps using the photo-conductivity method used in industrial Si solar cell production. A mask-less laser patterning with plasma etching of Si was tested.

## 2. Samples and Methods

Silicon wafers of a 〈001〉 surface orientation were coated with Al_2_O_3_ by electron-beam evaporation (Axxis, JKLesker). Femtosecond (fs) laser irradiation was implemented for the ablation of the holes in the mask for subsequent dry plasma etching of Si.

The femtosecond (fs) laser microfabrication setup based on the Pharos (Light Conversion) fs laser was integrated with scanning Aerotech xy-stages and SCA software control of laser radiation and scanning conditions (Workshop of Photonics). The pulse-to-pulse energy stability of the laser was ∼1% as monitored directly over a long ∼1 h time. This was important due to the sensitivity of the ablated hole’s diameter in the mask over a long laser writing time. Si samples were placed on a 10×10 cm^2^ porous ceramic chucking plate with a steel retainer with a flatness of 10 μm. This determined a well-defined sample plane, provided there were no micro-particulates between the sample and vacuum chuck, which can cause the bending of thin samples.

The plane approximating the sample’s surface was calculated using the three-point P${}_{1,2,3}$ method [21], where the coordinates of the focal point placed on the sample’s surface are determined. The plane through the point P${}_{1}$ (also P${}_{2}$ and P${}_{3}$) is given by $a_{n}\left(x-x_{1}\right)+b_{n}\left(y-y_{1}\right)+c_{n}\left(z-z_{1}\right)=0$ with the normal coefficients $\left(a_{n},b_{n},c_{n}\right)$ determined experimentally. This is the plane equation, which will account for the actual tilt, if any. During laser writing, for the each new irradiation point $P_{new}\left(x_{new},y_{new}\right)$ along the writing trajectory, the height $z_{new}$ is calculated from the plane equation given above. This accounted for the actual surface position. During writing over larger surface areas for a long time, the three-point method can be re-applied across smaller areas to keep the surface within the depth of focus.

Dry plasma etching was carried out using a reactive ion etching (RIE) tool (Samco) using an established recipe for PhC patterns: SF_6_:CHF_3_:O_2_ at a 5:1:1 flow rate ratio for 10–15 min. The inductively coupled plasma (ICP) power was 180 W with a 0 W radio frequency (RF) bias power for the Cr mask and 140 W and 5 W for the alumina mask.

Scanning electron microscopy (SEM) was used for the structural characterisation of the samples processed by the laser and plasma treatments (the Raith 150TWO electron beam writer was used in field-emission SEM mode).

The minority carrier lifetime was measured by the inductively coupled photo-conductance method with a Sinton 3000 (WCT-120), which is an established tool in the Si solar cell industry. The changes of the current $J_{ph}$ due to photo-induced carriers over time *t* after a Si sample/wafer was illuminated by a flash lamp were measured inductively and were read out as the voltage $V_{in}$ across a reference resistance. The measured voltage transient $J_{ph}\left(t\right)=fV_{in}\left(t\right)\left[{\mathrm{Const}}_{i}\times 38\;\mathrm{mA}/{\mathrm{cm}}^{2}\right]$, where *f* is the optical factor dependant on the optical properties of the wafer (surface texture, thickness, antireflection coating), ${\mathrm{Const}}_{i}$ is the constant determined by Sinton Instruments for individual testers, and the value of 38 mA/cm${}^{2}$ is the approximate short-circuit current density $J_{SC}$ of the reference cell under standard test conditions (the air mass AM 1.5 solar spectrum). Typical values of *f* = 0.67–0.73 are for a planar wafer with no antireflection coating, (0.80–0.95) for a planar wafer with an antireflection coating, and (0.95–1.15) for a textured wafer with an antireflection coating. For samples with the PhC texture, f=0.95 was used for all samples measured. An instantaneous lifetime determined from the measured $J_{ph}$ current transient is the minority carrier lifetime $\tau_{mc}$. It was used to trace the changes of the surface quality due to recombination during different processing steps.

Approximately 300 μm-thick and micro-thin (12 μm-thick) n- and p-type Si wafers were used in this study. A handling protocol was developed for the thin-Si wafers for cutting and patterning. A 4-inch Si wafer (fixed on a plastic film on a ring-mount) was placed onto a transparent 4-inch Al_2_O_3_ crystalline wafer (∼300 μm thickness) using ∼10 μm coatings of SU8 or AZ photoresists as a glue. After separation from the film, Si was cut into smaller pieces of arbitrary sizes as required for the experiments by fs laser dicing (irradiation from the Si side). The release of the Si cut-out chips was performed by placing the entire Si-Al_2_O_3_ sandwich into a developer. The organic developer was diluted with IPA rinse, and the floating Si chips were transferred from the rinse solution onto a desired substrate (e.g., cover or slide glass) for further fabrication and characterisation procedures.

## 3. Results and Discussion

### 3.1. Laser Ablation of Transparent Mask

**Figure 2:**
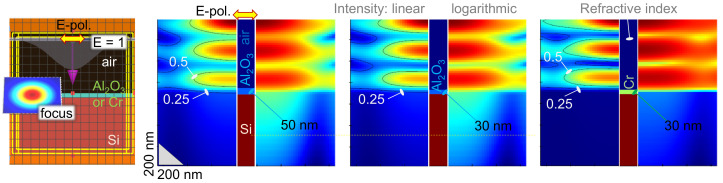
Numerical modelling of light focused onto a 700 nm diameter on the sample surface for a λ=515 nm wavelength. Linear *E* and lgE distributions are shown; the incident light E-field is E=1. Contours for E=0.5 and E=0.25 are shown. The central cross-section shows the refractive index distribution across the light propagation. The left panel shows the calculation volume in the finite difference time domain (FDTD) model (Lumerical, Ansys).

The numerical modelling of light intensity at the focal region was performed using the finite difference time domain (FDTD; Lumerical, Ansys). Figure 2 shows the light intensity distribution in the plane of incidence for focusing of a λ=515 nm wavelength into a 700 nm focal spot placed at the surface of the mask (0.65 μm from the light source, while the Si surface was at a 0.7 μm distance). This closely corresponds to the experimental conditions of mask ablation using an objective lense with a numerical aperture NA=0.9, i.e., a diameter at focus of 2r=1.22λ/NA=698 nm. The light distribution through the mask revealed that slightly less intensity was reflected and more was irradiated inside the Si for a thicker 50 nm alumina mask (see the horizontal dashed line in Figure 2). This was an effect of the partial antireflection coating due to the refractive index of alumina n=1.7 (used in FDTD). The antireflection coating for a single wavelength has to be a λ/4=129 nm thickness for 515 nm, and the refractive index $n_{arc}=\sqrt{n_{Si}}=1.87$. Figure 2 shows only a minor variation of the E-field intensity throughout the thickness of the mask (the |E| amplitude is plotted, while the intensity is $E^{2}$); for a comparison, a non-transparent 30 nm Cr mask is also shown. Apparently, the ablation of transparent films is affected by their antireflection action, similar as for membranes [22].

Despite laser ablation taking place at a high pulse intensity >1 TW/cm${}^{2}$ via nonlinear light–matter interaction, the linear optical properties such as the antireflection coatings play an important role. This increased the energy per pulse required to open an ablation hole in the alumina mask when it became thicker. In the case of 20 nm, as well as 50 nm masks, Si ablation caused hole opening in a transparent mask, and the brittle failure of Al_2_O_3_ was observed at near-threshold fluence rather than melting, as it was for the Cr mask.

**Figure 3:**
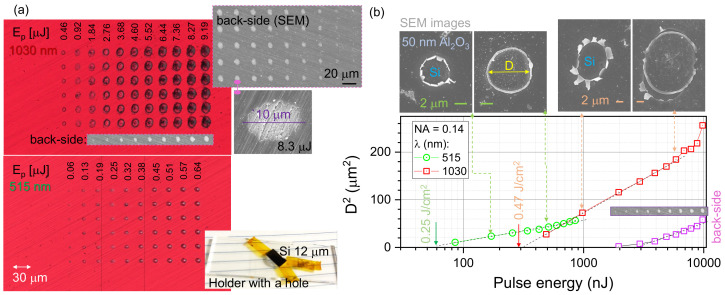
(**a**) Optical reflection image of single-pulse irradiated sites at NA=0.14 focusing on a 50 nm Al_2_O_3_-coated 12 μm-thick Si (inset photo) at different pulse energies $E_{p}$ (on the sample) and wavelengths λ=1030 nm and 515 nm. The SEM image insets show the back-side damage of the 12 μm-thick Si. (**b**) Ablation threshold of 50 nm-thick Al_2_O_3_ film on 12 μm-thick Si determined from the $D^{2}\propto \mathrm{lg}\left(E_{p}\right)$ dependence for two wavelengths λ=1030,515 nm at NA=0.14 focusing; $E_{p}$ was measured on the sample. The diameter of the back-side contrast change in the SEM image is plotted on the same $D^{2}$ dependence (for λ=1030 nm).

Next, we explored the power dependence of the hole ablation in a 50 nm-thick Al_2_O_3_ mask on a 12 μm-thick Si wafer via the standard $Diameter^{2}$$\left(D^{2}\right)$ power dependence (Figure 3; see Appendix B). Slightly different thresholds for λ=515 nm and 1030 nm were observed, and the fluence $F_{p}=0.25$ J/cm${}^{2}$ and 0.47 J/cm${}^{2}$, respectively. This is close to the Si ablation threshold at 0.2 J/cm${}^{2}$ for 515 nm, which is absorbed. The first harmonic 1030 nm laser pulses are in the spectral window of Si. At close to the threshold fluences, in both cases of λ, there was evidence of the brittle failure of the Al_2_O_3_ mask (see the SEM images in Figure 3b). The ablation of the Al_2_O_3_-coated Si was carried out in such a way that there was no back-reflected laser pulse for the 1030 nm case where the sample was transparent. A shorter 515 nm wavelength is better suited for mask ablation due to a lower slope of $D^{2}$ vs. $E_{p}$ dependence, which affords some leeway to make the same ablation hole size in the mask, regardless of small laser power fluctuations. Furthermore, there is no ns-pedestal emission from the laser due to the long excitation pulse from the laser diodes used as a pump source in a solid-state laser. This fs laser ablation surface texturing (FAST) was used to make the etch mask on the Si.

Interestingly, there were well-recognisable surface modification marks on the opposite side of the sample for the λ=1030 nm ablation (see the SEM inset in Figure 3a). The diameter of the laser-modified region was ∼3-times smaller than on the front surface (b). The difference of the slope (smaller) in the power dependence $D^{2}\propto E_{p}$ signifies a different nonlinear dissipation of the pulse energy over the propagation through the 12 μm-thick Si (see Section 3.4). The exit plane had the apparent ablation of nanoscale grooves on the Si back-surface, which were present on the Si surface (see the SEM inset in Figure 3a). The entire 12 μm thickness of the Si is inside the depth-of-focus or the double-Rayleigh length $2z_{r}=\pi r^{2}/\lambda \approx 62\;$μm.

**Figure 4:**
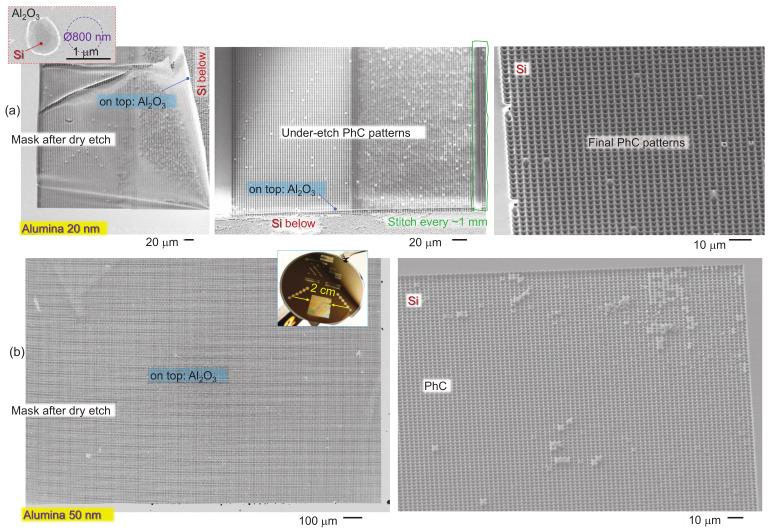
(**a**) SEM images of the Al_2_O_3_ mask (20 nm) after plasma etching and the final PhC pattern on Si. The middle panel shows the implemented strategy to introduce a few μm-wide bridges every 1 mm to prevent mask failure (left panel) due to under-etching. The pulse energy to open an ablation hole was $E_{p}=12.5$ nJ (on the sample). The inset shows the ablation opening in the mask, which matched exactly the focal diameter of 0.8 μm (before etching) close to the focal diameter 1.22λ/NA=700 nm. (**b**) A mask of 50 nm of Al_2_O_3_ (left image) maintained mechanical strength for the plasma etching over 1×1 cm${}^{2}$ areas. Si patterned using a 50 nm alumina mask after ultra-sonic mask removal (right image). Etched by SF_6_:CHF_3_:O_2_ at a 5:1:1 flow rate ratio for 10 min. The pulse energy to the open ablation hole was $E_{p}\approx 12$ nJ (on the sample). The inset shows the photo of the wafer-scale patterning tests used for the optimisation of the larger-area laser writing.

Figure 4 illustrates the experimental finding that 20 nm-thick alumina did not withstand the required 10–15 min plasma etch of the Si and failed mechanically. If the pattern on the mask was made in steps of 1 mm in one direction over the entire 1×1 cm${}^{2}$ area, the mask was mechanically robust enough to survive the plasma etching. No mechanical failure was observed when the mask was 50 nm thick, even for the entire 1×1 cm${}^{2}$ area.

### 3.2. Deep Etching of Si

**Figure 5:**
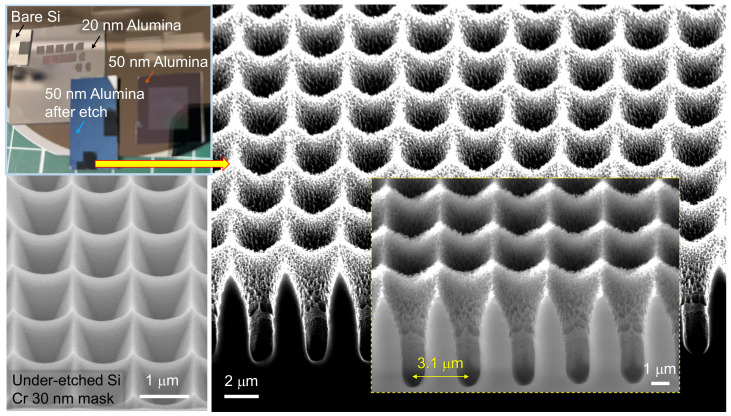
The colour photo shows Si samples with different Al_2_O_3_ masks before and after dry plasma etching. Left: the SEM image shows the Si PhC made by plasma etching developed for an under-etched zero-ridge tee-pee PhC pattern using a 30 nm-thick Cr mask [8]. Similar plasma etching conditions as used in the case of 30 nm of Cr showed very different tee-pee patterns for the 50 nm alumina mask ablated by $E_{p}\approx 4.2$ nJ pulses (at focus). Etched by SF_6_:CHF_3_:O_2_ at a 5:1:1 flow rate ratio for 15 min at ICP 180 W and 0 W bias (Cr mask) [8] and 30 min at ICP 140 W and 5 W bias (Al_2_O_3_ mask); He pressure of 2.70 kPa at a process pressure of 2.5 Pa.

Deeper structures were obtained by applying a small bias power of 5 W and decreasing the ICP power to 140 W. The ICP power is responsible for isotropic (all directional) etching, while direct bias enhances the directionality of etching normal to the surface. The total ICP and bias powers are usually kept close to constant, i.e., if the ICP is reduced, the bias can be increased. This was tested for the deeper etching (Figure 5). Plasma etching and ion sputtering have inherently strong dependences on the angle of incidence onto the surface. The fastest material removal is at 70–80${}^{\circ }$. This directional etching favours the formation of deeper holes with close-to-vertical walls, as observed experimentally. It is well established that isotropic Ar-plasma etching has very fast removal of fs-laser-modified regions [23]. This can be one of the reasons why deeper plasma-etched tee-pee patterns were fabricated. The Si etching used is slightly anisotropic, especially when used in the under-etching mode through small openings in the mask. This was judged from the tee-pee profiles, which still had a resemblance to the inverse pyramid patterns obtained in an anisotropic KOH wet etch. The typical aspect ratio of the depth/width≈1 for the wet and dry etch using 30 nm Cr and 20 nm alumina masks increased to ≈2. The thinning of the 50 nm alumina mask by etching was also recognisable by the colour change (see the colour photo in Figure 5 and Appendix A) in the non-patterned regions. Such thinning could contribute to the mechanical failure of thinner 20 nm masks under prolonged etching.

### 3.3. Mask-Less Etching of Si

**Figure 6:**
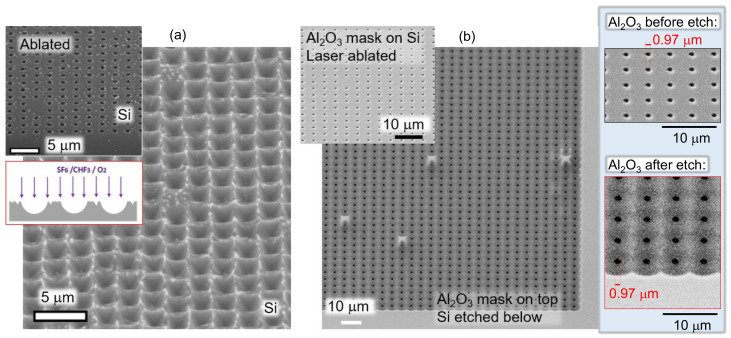
(**a**) Maskless patterning of Si with PhC light-trapping structures using fs laser ablation and dry plasma etching. Pulse energy $E_{p}\approx 3.2$ nJ (at focus), NA=0.9. The inset shows just the ablated surface of the Si. (**b**) Si patterning by plasma etching using a 20 nm-thick Al_2_O_3_ mask. $E_{p}=4.2$ nJ (at focus), NA=0.9. Left: The insets show close-up views of the laser-ablated openings in the mask, which have a diameter ∼1 μm for the 20 nm-thick Al_2_O_3_. The dry plasma etching conditions were the same as for the Al_2_O_3_ mask with the bias set at 5 W (a directional etch promoted).

Figure 6 compares two different PhC fabrication methods on the Si surface. When direct laser ablation by FAST was used without any mask to make the ablation pattern of the required periodicity on the surface, only $E_{p}$ = (1–2) nJ was required. When Si was ablated to open holes in a 20 nm-thick Al_2_O_3_ mask, an order of magnitude larger pulse energy >10 nJ was necessary. This was caused by the reflection change due to the coating (Figure 2), as well as the ablation pressure required to break through the alumina nano-film. The average intensity for $E_{p}=2$ nJ (on the sample) was $I_{p}=F_{p}/t_{p}=2.27$ TW/cm${}^{2}$ for fluence $F_{p}=E_{p}/\left(\pi r^{2}\right)=0.52$ J/cm${}^{2}$, where the radius of the focal spot r=0.61λ/NA=349 nm for NA=0.9, λ=515 nm, and pulse duration $t_{p}=230$ fs. This corresponds to approximately double the ablation threshold of Si for ultra-short sub-1 ps pulses, which is ∼0.2 J/cm${}^{2}$ [24].

The ablation threshold fluence $F_{th}$ [J/cm${}^{2}$] of Si and the Gaussian beam waist $w_{0}$ were experimentally established from the single-pulse ablation dependence of the diameter of the ablated crater $D_{a}$ vs. pulse energy $\ln E_{p}$: $D_{a}^{2}=2w_{0}^{2}\ln E_{p}$.

### 3.4. Energy Deposition and Nonlinear Light–Matter Interaction (above Ablation Threshold)

In order to test the conjecture of the structural modification of Si along the propagation of a fs laser pulse during mask writing, a separate experiment was carried out using a single-crystal 10 μm-thick Si wafer (orientation 〈001〉). By measuring the transmitted pulse energy vs. the incident light, a nonlinear light–matter interaction can be determined from a departure from the linear dependence. Measurements were carried out at lower NA=0.14 with a change of position on the sample for each incident pulse energy. When two-photon absorption (TPA) dominates (nonlinear absorption), the transmitted intensity through the sample of thickness *d* is $I_{t}\left(d\right)=\ln\left(1+I_{in}d\beta \right)/\left(d\beta \right)+Const$, where β (cm/W) is the TPA coefficient (the nonlinear intensity-dependent absorption coefficient $\alpha \left(I_{in}\right)=\alpha_{0}+\beta I_{in}$). This dependence is only valid when other nonlinear absorption mechanisms are absent, which is usually a valid assumption since TPA is the strongest among multi-photon absorption transitions. This dependence was found to describe fs laser polymerisation/printing well [25].

**Figure 7:**
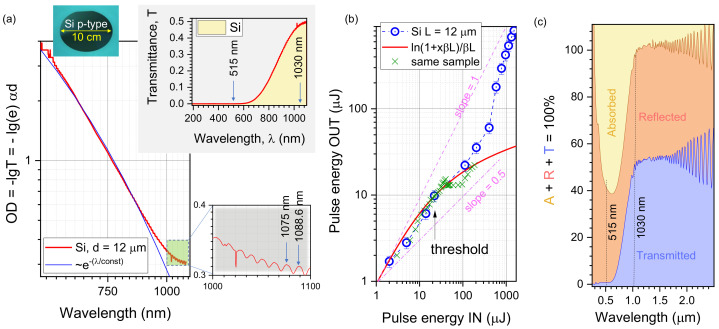
Transmission of micro-thin Si wafer. (**a**) Transmittance though d≈12 μm-thick Si wafer; note: log–log presentation for absorbance OD vs. λ. Thickness is determined from interference fringes: $\lambda_{1,2}$ are two adjacent maxima: $d=1/[2\left(1/\lambda_{1}-1/\lambda_{2}\right)n]$ with n=3.59 at a 1080 nm wavelength. (**b**) Transmittance at λ=1030 nm through the L=12 μm-thick Si wafer. Each irradiation point was refreshed using a pre-programmed sample scan during the measurement. Focusing was with an NA=0.14 objective lens into a focal spot of radius r=0.61λ/NA≈4.49 μm and a depth of focus $2z_{R}=\pi r^{2}/\lambda >d$. The single-pulse ablation threshold was recognisable (under the microscope) at $E_{p}^{IN}=22\;\mu$J. The fit by ln(1+xβL)/(βL) is shown with two-photon absorption coefficient β=858 cm/TW; *x* is the input pulse energy (intensity). The error bars are 15%. Cross markers show the data for the same sample and focusing conditions, only for the smaller step in energy between adjacent irradiation spots. (**c**) Portions (colour-coded) of the absorbed *A*, reflected *R*, and transmitted *T* power through the 12 μm-thick Si slab measured using an integrating sphere (Lambda 1050 UV/VIS/NIR spectrometer; PerkinElmer).

Figure 7a shows the transmittance $T=10^{-OD}\equiv e^{-\alpha d}$ data measured on a free-standing Si wafer. From the recognisable interference fringes at the longer side of the spectrum, the Fabry-Pérot etalon fringes are recognisable and correspond to the thickness of the Si slab d=12 μm (considering n=3.59 at around λ = 1 μm). The slab transmittance $T=\frac{{\left(n-1\right)}^{2}}{n^{2}+1}\approx 48.3\%$ and reflectance $R=\frac{2n}{n^{2}+1}\approx 51.7\%$ at normal incidence at the spectral region with weak absorption with n=3.59. Such values closely matched the experimental observation at the wavelengths close to the bandgap $E_{g}=1.12$ eV or $\lambda_{g}$ (μm) = $1.24/(E_{g}$ (eV)) = 1.11 μm. This implies that the absorption is negligible with κ→0; the absorption coefficient α=4πκ/λ. Strong absorption is defined as αd>1, and for d=10 μm, $\alpha >10^{3}$ cm${}^{-1}$.

The power dependence of transmittance was measured to explore the departure from the linear law, i.e., slope γ=1 between transmitted pulse energy $E_{p}^{OUT}$ vs. $E_{p}^{IN}$ at the transparency wavelength of λ=1030 nm (Figure 7b). When the only nonlinear losses in transmission are due to two-photon absorption (TPA), the first nonlinear absorption process, the transmitted power can be approximated by $I_{OUT}=I_{IN}\ln\left(1+I_{IN}\beta L\right)/\left(\beta L\right)$, where *L* is the interaction length and β is the TPA coefficient. This model was used to describe the energy deposition by an ultra-short laser pulse into a polymerisable resin [25]. In our case, the depth of focus or double-Rayleigh length $2z_{R}=\pi r^{2}/\lambda =61.5\;\mu$m was larger than the sample, and L=12 μm was used; the radius of the focal spot r=0.61λ/NA=4.5 μm. The transmitted power (pulse energy) was measured during a 10 s scan with refreshing the irradiation spot by ΔX=100 μm, which is larger than the focal diameter of ∼9 μm. A clear departure from the linear transmittance γ=1 was observed, and the TPA coefficient β=858 cm/TW. Interestingly, at higher pulse energies, the reverse trend was observed showing larger *T* returning to the linear γ=1 slope. Since the measurements were carried out for single pulses and the refreshed surface area for each laser shot, this transmittance increase reflects the induced transparency at high irradiance. The single-pulse ablation threshold was observed at $E_{p}^{IN}=22\;\mu$J, which corresponds to average fluence $F_{p}=34.8$ J/cm${}^{2}$/pulse and $I_{p}=F_{p}/t_{p}=151$ TW/cm${}^{2}$/pulse for pulse duration $t_{p}=230$ fs. This is more than two orders of magnitude larger than for the thick (≥100 μm) Si (0.2 J/cm${}^{2}$/pulse [24]). This is understandable due to the lack of absorbance *A* since reflectance *R* and transmittance *T* make R+T≈1 with a negligible portion of *A* at a wavelength of irradiation λ=1030 nm (Figure 7).

Figure 7c shows portions of the experimentally measured reflected and transmitted light power at different wavelengths. Only a few % of light were absorbed at λ=1030 nm, and a high portion of light ∼50% reflected over the transparency window in the near-IR due to the high real part of the refractive index of Si n≈3.6. The low absorbance caused the higher pulse energy required for laser ablation of transparent Al_2_O_3_ mask patterning. It is also a fundamental cause of poor light harvesting by thin solar cells. When a thicker 50 nm Al_2_O_3_ mask was used on the Si, the reflectivity was reduced due to the smaller refractive index contrast. However, a larger pulse energy is required for ablating a hole through a thicker transparent mask film. As a result, a deeper energy deposition takes place and causes the formation of high-aspect-ratio structures after plasma etching (Figure 5).

### 3.5. Minority Carriers’ Lifetime

One of the industry-accepted quality tests for solar cell performance is the measurement of the minority carrier lifetime. A short lifetime and high surface recombination velocity *S* limits the collection of photo-carriers outside the solar cell, hence reducing the efficiency of light-to-electricity power conversion. We used the Sinton WCT-100 instrument, which works via inductive detection of the total recombination currents in the bulk and on the surface. A light flash creates photo-excited carriers, electrons–hole pairs (e–h) with concentration Δn=Δp, which are much smaller than those due to doping (*n* or *p*). This is orders of magnitude higher in the solar cells p≈$10^{17}$ –$10^{18}$ cm${}^{-3}$ used for high efficiency including those with the IBC architecture. The diffusion coefficient of the minority carriers $D_{mc}$ (cm${}^{2}$/s) defines the surface recombination lifetime $\tau_{s}=\frac{4}{D_{mc}}{\left(\frac{W}{\pi }\right)}^{2}$ for one recombination active surface with high surface recombination velocity *S* (cm/s) (the other is perfectly passivated S=0), and *W* is the thickness of the cell. A general expression is $\tau_{s}=\frac{W}{2S}+\frac{1}{D_{mc}}{\left(\frac{W}{\pi }\right)}^{2}$ for both solar cell surfaces having the same $S=S_{1}=S_{2}$ on both surfaces. The first term $\frac{W}{2S}$ defines the surface recombination lifetime, and the second term $\frac{W^{2}}{\pi^{2}D_{mc}}$ is the time required for carrier diffusion from the bulk of the sample’s surface. These times are added together since the processes occur one after the other. In parallel, the bulk recombination also removes the carriers. The effective carrier lifetime is defined by the bulk and surface contributions $1/\tau_{eff}=1/\tau_{b}+1/\tau_{s}$. For the quasi-steady-state (QSS), $\tau_{eff}\gg \frac{W^{2}}{\pi^{2}D_{mc}}$.

For low doping concentrations $N_{D}\leq 7\times 10^{17}$ cm${}^{-3}$, S≈70 cm/s [26], the “geometrical” contribution to $\tau_{s}$, namely $\frac{W}{2S}=5\;\mu$s for W=10 μm-thick Si cell and $S=10^{2}$ cm/s. This estimate shows that Si solar cells of tens-of-micrometres in thickness need good passivation to reduce the surface losses of photo-generated carriers. *S* is defined $S=N_{ss}v_{th}\sigma$ by the surface state density $N_{ss}$ (cm${}^{-2}$) of recombination centres at the surface; the average thermal velocity $v_{th}=\sqrt{3k_{B}T/m^{*}}\approx 10^{7}$ cm/s (T=300 K), and σ (cm${}^{2}$) is the recombination cross-section of the recombination centre ($k_{B}$ is the Boltzmann constant; $m^{*}$ is the effective mass of the charge carrier; *T* is the absolute temperature).

**Figure 8:**
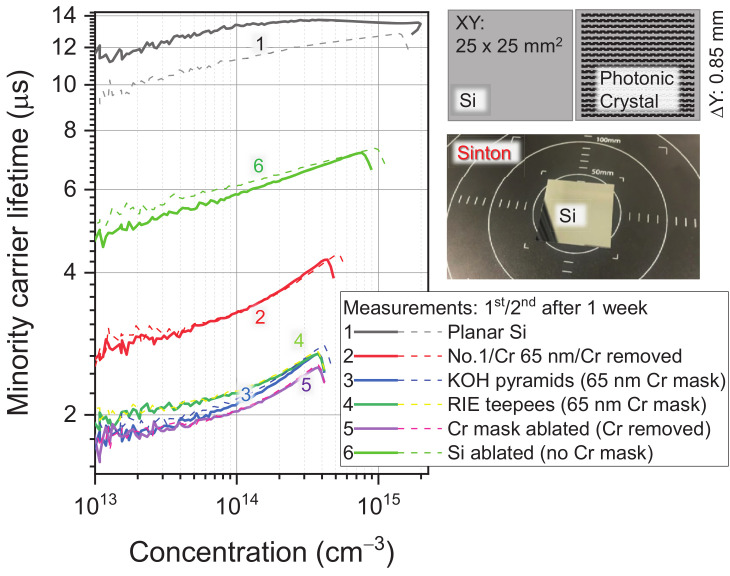
The lifetime of minority carriers $\tau_{mc}$ vs. the concentration in the Si with different surface treatments measured with the Sinton (Si p-type, boron doping). The inset shows the schematics of the fabricated patterns on the 25×25 mm${}^{2}$ area samples. Photonic crystal structures with a Λ=3.1 μm period were laser patterned with a Δy=0.85 mm separation (an ∼15% coverage of the surface by PhC was performed). The quasi-steady-state (QSS) mode of calculations was carried out with optical factors f=0.7 (non-textured, Nos. 1, 2) and f=0.95 (textured, Nos. 3, 4, 5, 6) for the samples. The Si wafer here was 300 μm thick, and the Cr film used for the mask was e-beam deposited.

Figure 8 summarises the lifetime measurements from the equivalent-area samples treated by different processing steps used in PhC texturing of Si for solar cell applications, e.g., Cr coating, Cr wet etch removal, Cr mask ablation, and KOH- and RIE-patterned Si surfaces of the final design after Cr mask removal. The lifetime of the as-received Si wafer was $\tau_{mc}∼12\;\mu$s, which is typical for a Czochralski-grown material 10–50 μs and is 50–150 μs for the float-zone Si [27]. It was revealed that the e-beam evaporation of the Cr mask and its subsequent removal already significantly reduced $\tau_{mc}$. Direct laser ablation of Si (without the mask) only reduced the lifetime by half. The final PhC patterns after wet KOH or dry plasma etching had the $\tau_{mc}$ typically reduced to 2 μs. These low values were caused by a significant contribution of the surface recombination. The surface recombination rate can be reduced by thermal treatments up to 450 °C, by passivisation and antireflection coatings applied for the actual Si solar cells. Here, we compared the evolution of $\tau_{mc}$ under the processing steps required for the PhC definition using different mask application, fabrication, and removal steps. It was encouraging to find that the laser ablation itself was not the main contributor to reducing the carrier lifetime values. This was expected since the Si volume removed by etching (wet or dry) was from the region directly irradiated by the laser during mask ablation. The volume most affected by the laser was removed during the formation of the PhC surface texture for light trapping. Interestingly, $\tau_{mc}$ measured from the Si surface just after the ablation of the holes (without the Cr mask, as in the inset of Figure 6a) showed slightly larger $\tau_{mc}$ values after one week at room conditions, most probably due to passivisation by oxidation. All the $\tau_{mc}$ measurements were carried out on the same-sized samples. In a separate experiment, we established that $\tau_{mc}$ can significantly vary up to a factor of 2–3 just because of the sample size (with respect to the size of the inductive coil-reader in the centre of the measuring pad).

## 4. Conclusions and Outlook

The light trapping at the near-bandgap wavelengths of Si of ∼1 μm can be enhanced using PhC-textured Si surfaces. The fabrication of such PhC patterns using a dielectric transparent Al_2_O_3_ mask was made at different thicknesses, 20 and 50 nm. Such masks are promising due to the better surface quality and avoidance of metallic masks, e.g., Cr, which causes a reduced lifetime of the minority carriers, leading to a reduced efficiency of the eventual Si solar cell. Furthermore, direct patterning of the Si surface by ablation (even without a dielectric mask) can be performed for plasma etching of PhC patterns. It was found that a thicker Al_2_O_3_ mask caused the fabrication of higher-aspect-ratio structures (deeper holes). This was caused by the reduced reflectivity of the surface due to the Al_2_O_3_ coating and the larger pulse energy required to open a hole in the mask. The holes in the Al_2_O_3_ mask did not show melting signatures as compared with the Cr mask used earlier [8]. The minority carriers’ lifetime tests showed the strong dependence of the $\tau_{mc}$ values on the surface treatments, which are required for the PhC patterning of light-trapping surfaces.

The analysis of the 12 μm-thick Si transmission and energy deposition during the fs laser patterning revealed the presence of an optical nonlinearity (departure from the linear transmittance at slope=1), which was directly measured by virtue of the small thickness. The upper bound of the TPA for Si at λ=1030 nm was estimated as β=858 cm/TW. This high effective value was affected by thickness of the Si, where reflections from both surfaces contributed to *T* and *R* (hence, also A≡1−R−T).

## Figures and Tables

**Figure A1:**
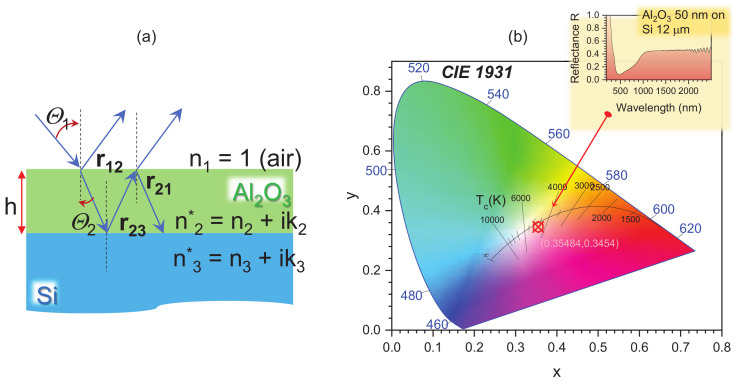
(**a**) Schematics and conventions for reflection coefficient *r* (Equation (A1)) from a thin film (Al_2_O_3_) of thickness *h* on Si; reflectance $R\equiv {\left|r\right|}^{2}$, and $n_{i}^{*}$ is the complex refractive coefficient of the *i*th layer. (**b**) Chromaticity plot for reflectance *R* from a 50 nm Al_2_O_3_-coated 12 μm Si film (inset shows spectrum R(λ)). The marker points to the (x,y)=(0.35484,0.3454) point.

## Data Availability

Data available upon request.

## Appendix A. Reflection Colour

The colour change of a Al_2_O_3_ mask after plasma etching indicated a change of thickness *h*. The reflection coefficient for the E-field for a thin film on a highly reflective substrate is given by [28]

(A1) $$r=\frac{r_{12}+r_{23}e^{2i\beta }}{1+r_{12}r_{23}e^{2i\beta }},$$

where $\beta =\frac{2\pi }{\lambda }n_{2}^{*}h\cos\theta_{2}$, $\theta_{2}$ is the refraction angle, and $r_{ij}$ is the polarisation-dependent Fresnel reflection coefficient for the angle of incidence $\theta_{1}$ (integers i,j=1…3 mark Ambient 1 (air), 2 (Al_2_O_3_), and 3 (Si)). Figure A1a shows the different contributions to reflectance, which cause the colour appearance of the film, e.g., the Al_2_O_3_ mask. Reflectance *R* (Figure A1b) of such a film is mapped onto (x,y) colour coordinates on the chromaticity map (380–780 nm visible colours).

Alumina has an amphoteric nature and can be dissolved in basic KOH wet etch, as well as acidic, e.g., HF-based, solutions. The colour change provides sensitive feedback for mask changes in dry (Figure 5) and wet etching.

## Appendix B. Focal Spot Size: Definitions

It is convenient to define the focal spot size by the Airy disk, i.e., the first minimum of the focused plane wave as $1.22\lambda /NA=2.44\lambda f_{\#}$, where the f-number $f_{\#}\equiv F/D$ and numerical aperture $NA=n\sin\left[\arctan\left(\frac{1}{2f_{\#}}\right)\right]\approx n\frac{1}{2f_{\#}}$, which is valid as long as sinα≈tanα or $f_{\#}>1.2$ (fast focusing is at smaller $f_{\#}$); *D* is the diameter of the beam/aperture; *F* is the focal length; *n* the refractive index at focus. The Airy disk contains 86% of the laser pulse/beam energy. This is comparable with a transmitted power of the Gaussian pulse/beam through the aperture, which has a radius of the beam waist $1-e^{-2}=86.5\%$. When the laser beam is expanded and only the central part is entering the objective lens (clipped on the entrance pupil aperture), the intensity at the focus resembles the Airy pattern with a ring; however, when the diameter of a Gaussian pulse/beam closely matches the entrance pupil, the intensity at the focus is Gaussian-like with radial profile $I\left(r\right)=I_{0}e^{-2r^{2}/w_{0}^{2}}$, where $w_{0}$ is the waist (radius).

For the Gaussian beam focused with a lens of $f_{\#}=F/D=1/\left(2NA\right)$, the focal spot is $2w_{0}=\frac{4\lambda }{\pi }\times \frac{F}{D}=\frac{4\lambda }{\pi }f_{\#}=\frac{4\lambda }{\pi }\times \frac{1}{2NA}$ (in air) and the depth-of-focus where the beam is expanding by a $\sqrt{2}$ factor is $DoF=\frac{8\lambda }{\pi }\times {\left(\frac{F}{D}\right)}^{2}$, for example with NA=0.7 and a λ=515 nm waist $w_{0}=234$ nm (Gaussian) or $r_{f}=0.61\lambda /NA=449$ nm (Airy disk). The Gaussian beam intensity is more strongly localised than the central Airy disk. For the tight (fast) focusing when the validity of sinα=tanα breaks, e.g., at NA=0.9, a direct estimate of the beam waist could be obtained from the slope of the linear fit of the ablation pit diameter on the pulse energy $D_{a}^{2}=f\left(\ln\left(E_{p}\right)\right)$:

(A2) $$D_{a}^{2}=2w_{0}^{2}\left(\ln\left(E_{p}\right)+\ln\left[\frac{2}{\pi w_{0}^{2}F_{th}}\right]\right),$$

where $F_{p}=E_{p}/\left(\pi w_{0}^{2}\right)$ is the fluence ($F_{th}$ is the threshold fluence when D→0).

This approach also accounts for the beam quality factor $M^{2}$, which is 1 for the ideal Gaussian pulse, but can have a range of values in 1.1–1.5 for the actual laser beam/pulse. This experimental dependence also defines the threshold ablation value at the cut-off value of D→0. For the Gaussian pulse/beam, the average intensity $I_{p}=E_{p}/\left(\pi w_{0}^{2}t_{p}\right)$ is half of the peak intensity $I_{0}=2I_{p}$. The full angular width of diverging angle after the focus is $\theta_{d}=D/F=1/f_{\#}$ for the Gaussian beam. The full-width at half-maximum (FWHM) of the Gaussian intensity profile is related to the waist $FWHM=\sqrt{2\ln2}w_{0}\approx 1.18w_{0}$. The intensity of $0.5I_{p}$ is at $0.59w_{0}$, $I_{p}/e$ at $0.71w_{0}$, and $I_{p}/e^{2}$ (13.5% level) at $w_{0}$.
